# Nomogram for predicting early death in elderly patients with laryngeal squamous cell carcinoma: A population-based SEER study

**DOI:** 10.1371/journal.pone.0315102

**Published:** 2024-12-19

**Authors:** Qi-Wei Liang, Xi-Lin Gao, Jun-wei Zhang

**Affiliations:** 1 Department of Otorhinolaryngology of Longgang Center Hospital, The Ninth People’s Hospital of Shenzhen, Shenzhen, China; 2 Department of Gastroenterology of Longgang Center Hospital, The Ninth People’s Hospital of Shenzhen, Shenzhen, China; Tehran University of Medical Sciences, ISLAMIC REPUBLIC OF IRAN

## Abstract

**Background:**

The disease and mortality rates of patients with laryngeal squamous cell carcinoma (LSCC) stabilize after peaking at the age of 60 years. This study aimed to identify risk factors associated with early death (death within 6 months) in elderly (≥60 years) patients with LSCC and to establish predictive nomograms to aid clinicians in developing individualized treatment plans.

**Methods:**

Data pertaining to elderly patients with LSCC between 2004 and 2015 was obtained from the Surveillance, Epidemiology, and End Results database (version 8.4.0). Multiple logistic models were used to identify the independent risk factors associated with early mortality. The overall risk of early death was predicted using a web-based probability calculator and predictive nomogram. The cohort underwent decision curve analysis (DCA), calibration, and receiver operating characteristic curves to evaluate the clinical applicability and predictability of the models during the training and validation stages.

**Results:**

This study included 10,031 patients, of which 1,711 (17.0%) experienced all-cause early death, and 1,129 died from cancer-specific causes. Patients with LSCC who had overlapping laryngeal lesions, advanced age, unmarried status, high tumour and node stages, presence of distant metastases, and lack of treatment were at risk for early death. According to the nomograms, the risk of all-cause death and cancer-specific early death had an area under the curve of 0.796 and 0.790, respectively. Internal validation and DCA revealed that the prediction model was accurate and could be applied clinically.

**Conclusion:**

The study provides an overview of the characteristics of early death in patients with LSCC. Among the prognostic factors, T stage and radiotherapy demonstrated the strongest predictive value for early mortality, while marital status and tumor grade had the worst prognostic value. Two nomogram plots were constructed to facilitate accurate prediction of all-cause and cancer-specific early mortality within 6 months in elderly patients with LSCC, thereby helping clinicians in providing more personalised treatment plans.

## Introduction

Laryngeal carcinoma, one of the most prevalent head and neck cancers, accounts for approximately 25% of all head and neck cancer cases [1]. Histopathologically, laryngeal squamous cell carcinomas (LSCCs) account for approximately 90% of laryngeal malignancies [2]. Global estimates for 2020 projected approximately 184,615 new cases of LSCC and 99,840 deaths worldwide due to this condition. LSCC accounts for approximately 1.5% of all new cancer cases, with an age-standardized incidence rate of approximately 3.6, and its mortality rate accounts for approximately 0.5% of all-cancer mortality [3]. The unique location of the laryngopharynx affects essential human functions such as swallowing, breathing, and vocal functions. Moreover, the larynx is surrounded by vital structures such as blood vessels and nerves. Therefore, patients with LSCC might be susceptible to increased mortality risks owing to the rapid local tumour progression [1, 4].

The prevalence of head and neck cancers among individuals aged ≥60 increased by approximately 45% between 1995 and 2015, primarily due to the increasing ageing population [5]. The incidence, prevalence, and mortality rates of LSCC increase with age, with a peak occurring after 60 years of age, followed by a plateau [6]. Over the past 20 years, LSCC has demonstrated a declining survival rate, particularly in elderly patients and those with advanced-stage laryngeal cancer [6, 7]. The development of LSCC is largely attributed to long-term smoking and drinking, and the early symptoms of laryngeal cancer are vague and unclear, leading to delayed diagnosis [8–10]. At an older age, due to the growth and spread of local tumour, a final diagnosis can then be made. This will inevitably reduce the effectiveness of treatment and lead to more unfavorable outcomes [11]. Additionally, elderly patients with LSCC often have poor physical or nutritional conditions, making them more susceptible to complications and mortality during treatment compared with younger patients, eventually resulting in early death [12]. Early death among elderly patients might significantly contribute to the decline in the survival rate of LSCC. Although age, tumor site, grade, and tumor stage are considered the main prognostic factors for LSCC, they are not sufficient to predict early death in elderly patients, especially since their prognostic contributions have not been determined. Few studies have specifically analysed the clinical risk factors associated with early death in elderly patients with LSCC, and there is a lack of distinction regarding high-risk patients. As a result, clinicians face challenges in effectively screening and identifying these patients and providing timely interventions.

While the American Joint Committee on Cancer (AJCC) tumour, node, and metastasis (TNM) staging system offers a fundamental assessment of prognosis for patients with LSCC, it lacks the ability in predicting early death among older patients due to the exclusion of several prognostic factors in the TNM staging.

Hence, this study aimed to use the Surveillance, Epidemiology, and End Results (SEER) database to analyse the prognostic factors associated with LSCC in elderly patients. Additionally, our objective was to develop a predictive model for early death in elderly patients with LSCC, thereby aiding clinicians in identifying elderly patients who are at high risk for early death, enabling them to devise individualized treatment strategies and shorten the time between diagnosis and treatment initiation.

## Methods

### Ethics statement

This study was approved by Longgang Center Hospital. The need for informed patient consent was waived because the data obtained from the SEER database are de-identified patient data. All analyses conducted in this study were in accordance with the 1964 Declaration of Helsinki and its later amendments or comparable ethical standards.

### Data collection

The SEER database, recognized as one of the most authoritative cancer databases globally, comprises data from 18 population-based cancer registries representing approximately 28% of the United States population [13]. For this study, data were obtained from the SEER database using the SEER * stat software (version 8.4.0). The dataset included patients diagnosed with LSCC between 2004 and 2015. The database version used in this study was the November 2019 release, known as Incidence-SEER Research Plus Date, 17 Registries, November 2021 Sub (2000–2019).

**Fig 1:**
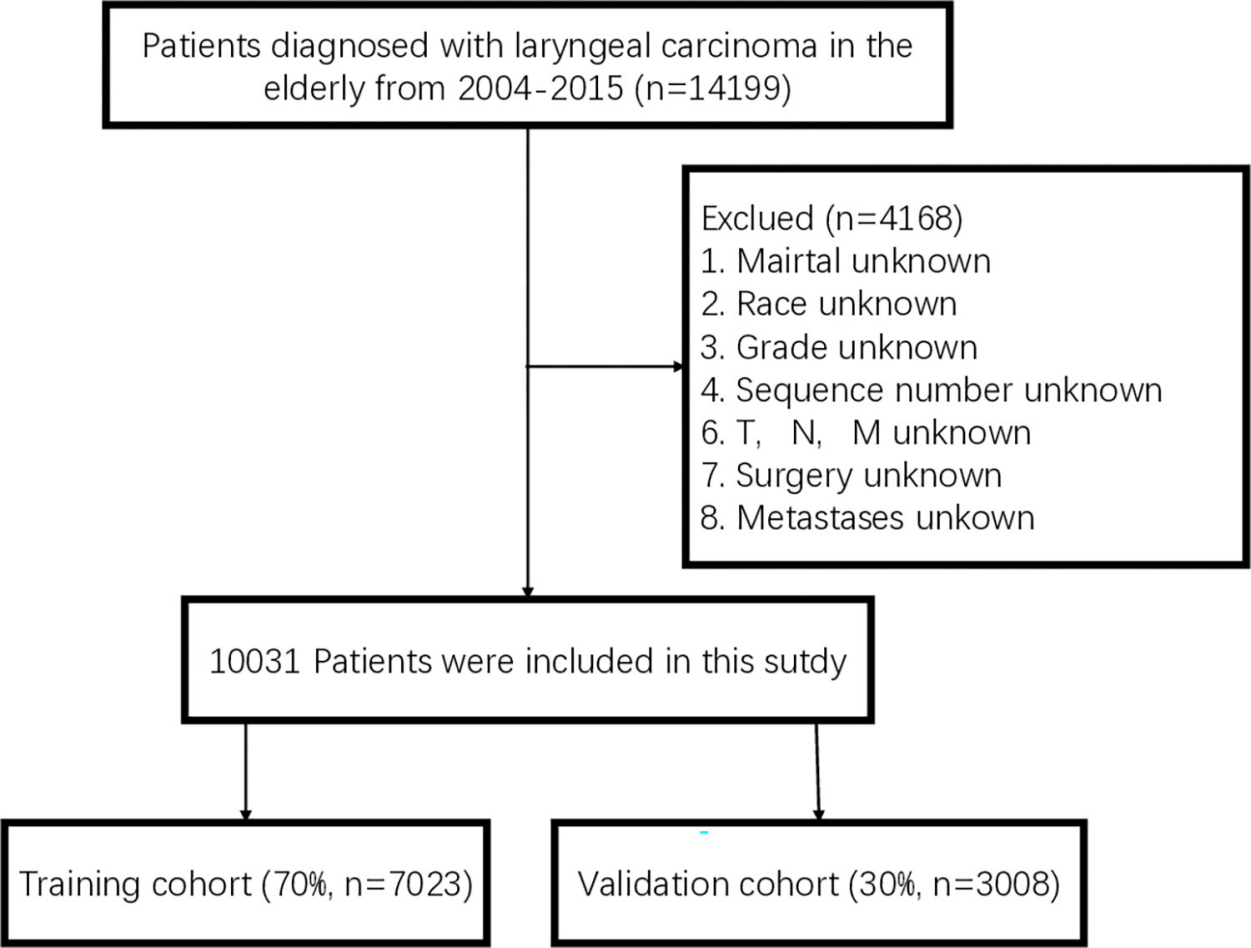
Flowchart of data selection of the elderly LSCC patients.

Due to the limited availability of chemotherapy data in the SEER database prior to 2004, this study focused on elderly patients diagnosed with LSCC between 2004 and 2015. Herein, patients aged ≥60 years were considered elderly, as there is no universally accepted definition for this age group [7, 14]. The primary site of LSCC was determined using the International Classification of Oncology, Third Edition criteria (C32.0-C32.9). Relevant information, including race, year of diagnosis, marital status, age, sex, TNM staging (AJCC, sixth edition), pathological classification, previous cancer history, and treatment details (surgery, radiotherapy, and chemotherapy), was retrieved from the SEER database. Exclusion criteria encompassed cases with unknown marital status, unknown race, and missing information on grade, sequence number, TNM stage, surgery, and metastases. Finally, 10,031 patients were included in the study, and the screening process is illustrated in Fig 1. A prediction model was developed and validated using a random allocation of patients in a 7:3 ratio in the training and validation cohorts [15]. The clinical data obtained included the age at diagnosis, race, sex, grade, primary site, the TNM stage, sequence number, cause of death, chemotherapy, surgery, radiotherapy, survival months, and vital status. Cancer-specific deaths were defined as cases where the SEER database indicated "dead of this cancer" as the cause of death, while all other deaths were considered non-cancer-specific deaths.

### Statistical analyses

There is currently no universally accepted definition for early death in the context of this study. The median survival for LSCC among the elderly patients in our study was 28 months. In our study, early death was defined as a survival time of ≤6 months. The prognostic factors associated with early death were identified using unit univariate and unit multivariate logistic regression analyses. Variables that have an impact on the prognosis of laryngeal cancer patients in previous literature were screened from the SEER database and included in this study. Baseline variables that were clinically relevant or had a univariate relationship with the outcome (P value <0.05) were entered into the unit multivariate logistic proportional hazards regression model. Nomograms were constructed based on the selected variables from the logistic regression model for predicting all-cause and cancer-specific early death. The predictive performance of the nomograms was evaluated in the training and validation cohorts using the receiver operating characteristic curve and the area under the curve (AUC). The nomogram’s predicted probabilities were compared with the actual outcomes using bootstrapping with 1,000 resamples. Additionally, the clinical applicability of the nomograms was quantified using decision curve analysis (DCA). The “DynNom” and “shiny” packages provided dynamic predictions of the likelihood of death.

Statistical analysis was performed using the R programme (version 3.6.2). Statistical significance was set at P <0.05 (two-tailed value).

## Results

### Baseline characteristics and incidence of early death

In this study, data on 10,031 elderly patients diagnosed with LSCC between 2004 and 2015 were extracted from the SEER database. The baseline characteristics of the patients reveals that most patients were white (83.1%, n = 8,340), male (80.8%, n = 8,103), married (52.5%), and had a history of previous cancer (26.8%). Among the 10,031 elderly patients with LSCC, 1,711 (17.0%) experienced all-cause death, 1,129 (11.2%) had cancer-specific deaths, and 582 experienced non-cancer-specific deaths (Table 1). The baseline characteristics of the training cohort consisting of 7,023 patients and the validation cohort consisting of 3,008 patients are included in S1 Table.

**Table 1:** Baseline characteristics of elderly patients diagnosis with advanced laryngeal carcinomas with or without early death.

Characteristic	Number of patients (%)			
	Overall N = 10031	No early death N = 8320	All-cause early death N = 1711	Cancer- specific early death N = 1129
Age				
60–69	4318 (43.0)	3665 (44.1)	653 (38.2)	417 (36.9)
70–79	3641 (36.3)	3037 (36.5)	604 (35.3)	395 (35.0)
≥80	2072 (20.7)	1618 (19.4)	454 (26.5)	317 (28.1)
Gender				
Male	8103 (80.8)	6759 (81.2)	1344 (78.6)	871 (77.1)
Female	1928 (19.2)	1561 (18.8)	367 (21.4)	258 (22.9)
Race				
White	8340 (83.1)	6950 (83.5)	1390 (81.2)	923 (81.8)
Black	1356 (13.5)	1082 (13.0)	274 (16.0)	173 (15.3)
Others/Unknown	335 (3.3)	288 (3.5)	47 (2.7)	33 (2.9)
Marital status				
Married	5266 (52.5)	4540 (54.6)	726 (42.4)	463 (41.0)
Unmarried	4765 (47.5)	3780 (45.4)	985 (57.6)	666 (59.0)
Primary site				
Glottis	4946 (49.3)	4386 (52.7)	560 (32.7)	348 (30.8)
Supraglottis	3849 (38.4)	3035 (36.5)	814 (47.6)	544 (48.2)
Subglottis	206 (2.1)	167 (2.0)	39 (2.3)	28 (2.5)
Overlapping lesion of larynx	328 (3.3)	250 (3.0)	78 (4.6)	53 (4.7)
Larynx, NOS	702 (7.0)	482 (5.8)	220 (12.9)	156 (13.8)
Prior cancer history				
Yes	7339 (73.2)	6111 (73.4)	1228 (71.8)	847 (75.0)
No	2692 (26.8)	2209 (26.6)	483 (28.2)	282 (25.0)
Grade				
Ⅰ/II	7493 (74.7)	6327 (76.0)	1166 (68.1)	756 (67.0)
Ⅲ/V	2538 (25.3)	1993 (24.0)	545 (31.9)	373 (33.0)
T stage				
T1	3556 (35.5)	3239 (38.9)	317 (18.5)	181 (16.0)
T2	2595 (25.9)	2177 (26.2)	418 (24.4)	260 (23.0)
T3	2193 (21.9)	1679 (20.2)	514 (30.0)	353 (31.3)
T4	1687 (16.8)	1225 (14.7)	462 (27.0)	335 (29.7)
N stage				
N0	7268 (72.5)	6246 (75.1)	1022 (59.7)	641 (56.8)
N1	1045 (10.4)	793 (9.5)	252 (14.7)	175 (15.5)
N2	1595 (15.9)	1211 (14.6)	384 (22.4)	271 (24.0)
N3	123 (1.2)	70 (0.8)	53 (3.1)	42 (3.7)
M stage				
M0	9646 (96.2)	8105 (97.4)	1541 (90.1)	994 (88.0)
M1	385 (3.8)	215 (2.6)	170 (9.9)	135 (12.0)
Surgery				
No	6247 (62.3)	4943 (59.4)	1304 (76.2)	875 (77.5)
Yes	3784 (37.7)	3377 (40.6)	407 (23.8)	254 (22.5)
Chemotherapy				
No/unknown	2594 (25.9)	1629 (19.6)	965 (56.4)	640 (56.7)
Yes	7437 (74.1)	6691 (80.4)	746 (43.6)	489 (43.3)
Radiotherapy				
No/Unknown	6925 (69.0)	5644 (67.8)	1281 (74.9)	829 (73.4)
Yes	3106 (31.0)	2676 (32.2)	430 (25.1)	300 (26.6)

### Identifying independent risk factors for early death

Univariate and multivariate logistic regression models were used to further investigate the risk factors for early mortality in elderly patients with LSCC. According to the univariate and multivariate logistic model analyses, age, primary site, grading, marital status, TNM stage, radiotherapy, surgery, and chemotherapy were associated with all-cause and cancer-specific early mortality (S2 Table). Patients aged ≥80 years (for all- cause death, OR 1.80 CI 1.49–2.18, p<0.001; for cancer-specific death, OR 1.82 CI 1.46–2.26, p<0.001), those who were unmarried (for all-cause death, OR 1.32 CI 1.14–1.52, p<0.001; for cancer-specific early death, OR 1.27 CI 1.08–1.50, p<0.001), individuals with grade III/IV disease (for all-cause death, OR 1.21 CI 1.03–1.42, p p = 0.017; for cancer-specific early death, OR 1.19 CI 0.99–1.43, p = 0.060), those with a history of TNM stages of T4 (for all-cause death, OR 3.62 CI 2.87–4.56, p<0.001; for cancer-specific early death, OR 4.16 CI 3.17–5.47, p<0.001), N3 (for all-cause death, OR 3.38 CI 2.05–5.59, p<0.001; for cancer-specific death, OR 3.47 CI 2.05–5.85, p<0.001), and M1 (for all-cause death, OR 1.76 CI 1.31–2.37, p<0.001; for cancer-specific death, OR 2.11 CI 1.56–2.87, p<0.001), and patients who did not receive chemotherapy (for all-cause death, OR 2.04 CI 1.72–2.50, p<0.001; for cancer-specific death, OR 1.78 CI 1.45–2.22, p<0.001), radiation (for all-cause death, OR 5.55 CI 4.76–6.67, p<0.001; for cancer-specific death, OR 4.54 CI 3.70–5.26, p<0.001), or surgery (for all-cause death, OR 4.17 CI 3.44–5.01, p<0.001; for cancer-specific death, OR 4.01 CI 3.23–4.76, p<0.001) had a higher risk of experiencing all-cause and cancer-specific early mortality (Table 2).

**Table 2:** Multivariate logistic regression for analyzing the risk factors for early death.

Variable	All-cause early death			Cancer-specific early death		
	OR	95%CI	P value	OR	95%CI	P value
Age						
60–69	reference			reference		
70–79	1.12	0.95–1.32	0.188	1.18	0.97–1.43	0.097
≥80	1.80	1.49–2.18	**<0.001**	1.82	1.46–2.26	**<0.001**
Marital status						
Married	reference			reference		
Unmarried	1.32	1.14–1.52	**<0.001**	1.27	1.08–1.5	**0.005**
Primary site						
Glottis	reference			reference		
Supraglottis	1.50	1.14–1.52	**<0.001**	1.42	1.16–1.74	**0.001**
Subglottis	1.32	1.26–1.79	**<0.001**	1.66	0.97–2.83	0.064
Overlapping lesion of larynx	1.56	0.74–2.04	0.419	1.47	0.98–2.2	0.064
Larynx, NOS	1.91	1.08–2.23	**0.017**	1.81	1.36–2.41	**<0.001**
Grade						
Ⅰ/II	reference			reference		
Ⅲ/V	1.21	1.03–1.42	**0.019**	1.19	0.99–1.43	0.060
T stage						
T1	reference			reference		
T2	1.74	1.41–2.15	**<0.001**	1.93	1.49–2.5	**<0.001**
T3	3.03	2.44–3.75	**<0.001**	3.25	2.51–4.2	**<0.001**
T4	3.62	2.87–4.56	**<0.001**	4.16	3.17–5.47	**<0.001**
N stage						
N0	reference			reference		
N1	1.61	1.29–2.01	**<0.001**	1.54	1.2–1.99	**0.001**
N2	1.49	1.21–1.83	**<0.001**	1.43	1.13–1.81	**0.003**
N3	3.38	2.05–5.59	**<0.001**	3.47	2.05–5.85	**<0.001**
M stage						
M0	reference			reference		
M1	1.76	1.31–2.37	**<0.001**	2.11	1.56–2.87	**<0.001**
Surgery						
No	reference			reference		
Yes	0.24	0.2–0.29	**<0.001**	0.25	0.21–0.31	**<0.001**
Chemotherapy						
No/unknown	reference			reference		
Yes	0.49	0.4–0.58	**<0.001**	0.56	0.45–0.69	**<0.001**
Radiotherapy						
No/Unknown	reference			reference		
Yes	0.18	0.15–0.21	**<0.001**	0.22	0.19–0.27	**<0.001**

### Construction and assessment of the nomogram

**Fig 2:**
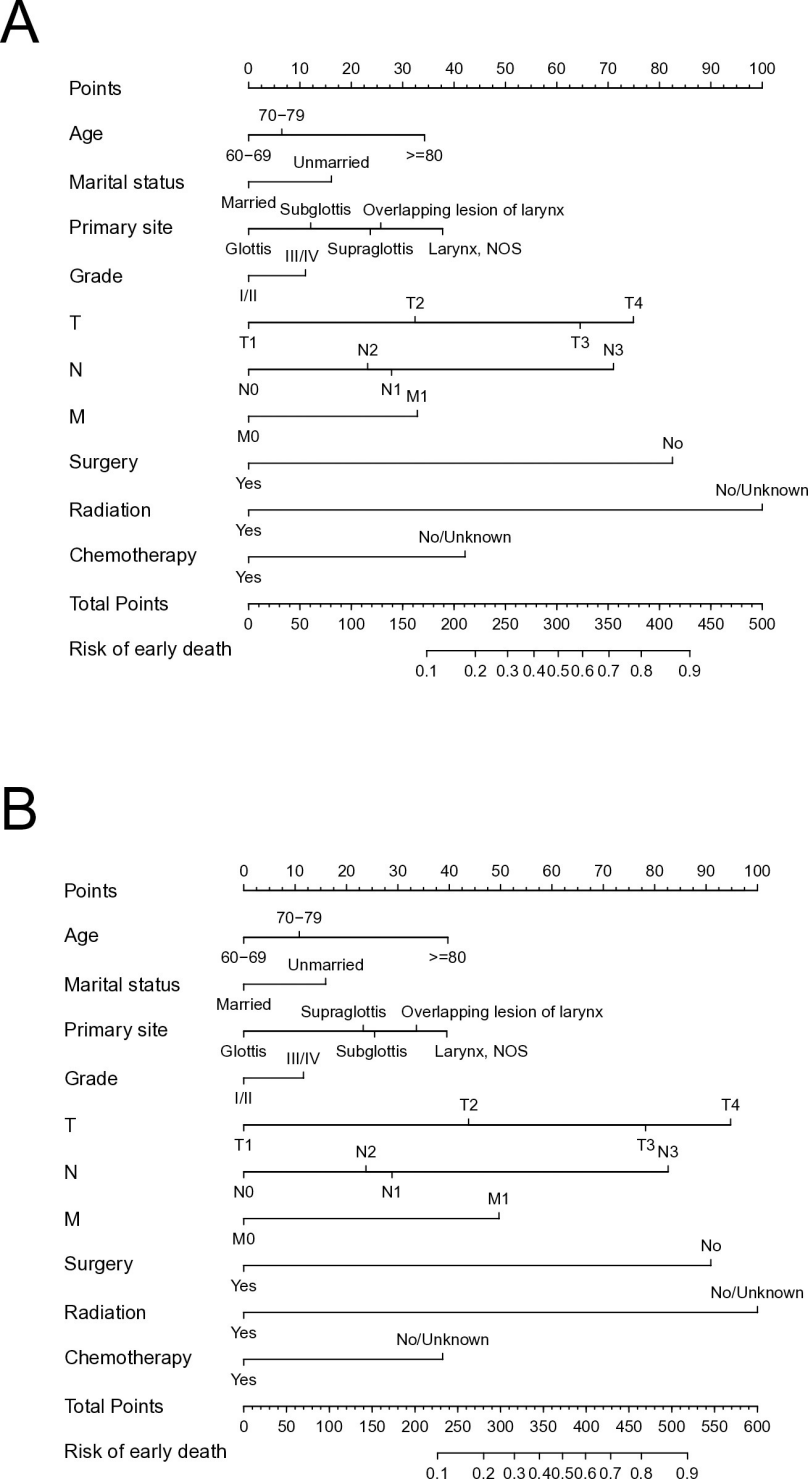
Nomograms for predicting all-cause (A) and cancer-specific early death (B) in elderly LSCC patients.

The multivariate logistic model in the SEER cohort yielded prediction nomogram plots for all-cause and cancer-specific early mortality risk variables, as illustrated in Fig 2A and 2B. These plots enable the calculation of prognostic factor scores in the predictive models. The variables included in both nomograms exhibited similar distributions, with only a slight difference in scores. The final score in the prediction model was obtained by adding the estimated points for each variable, thereby estimating the probability of early mortality in elderly patients with LSCC. Assuming a 75-year-old married male patient with T4N3M0 supraglottic laryngeal cancer, who only received chemotherapy and had a poorly differentiated tumour, the estimated risk of cancer-specific early death would be approximately 61%. The probability of all-cause and cancer-specific early mortality varied from 0.1–0.9. Among the prognostic factors, the T stage and radiotherapy demonstrated the strongest predictive value for early mortality, while marital status and tumour grade had the worst prognostic value.

**Fig 3:**
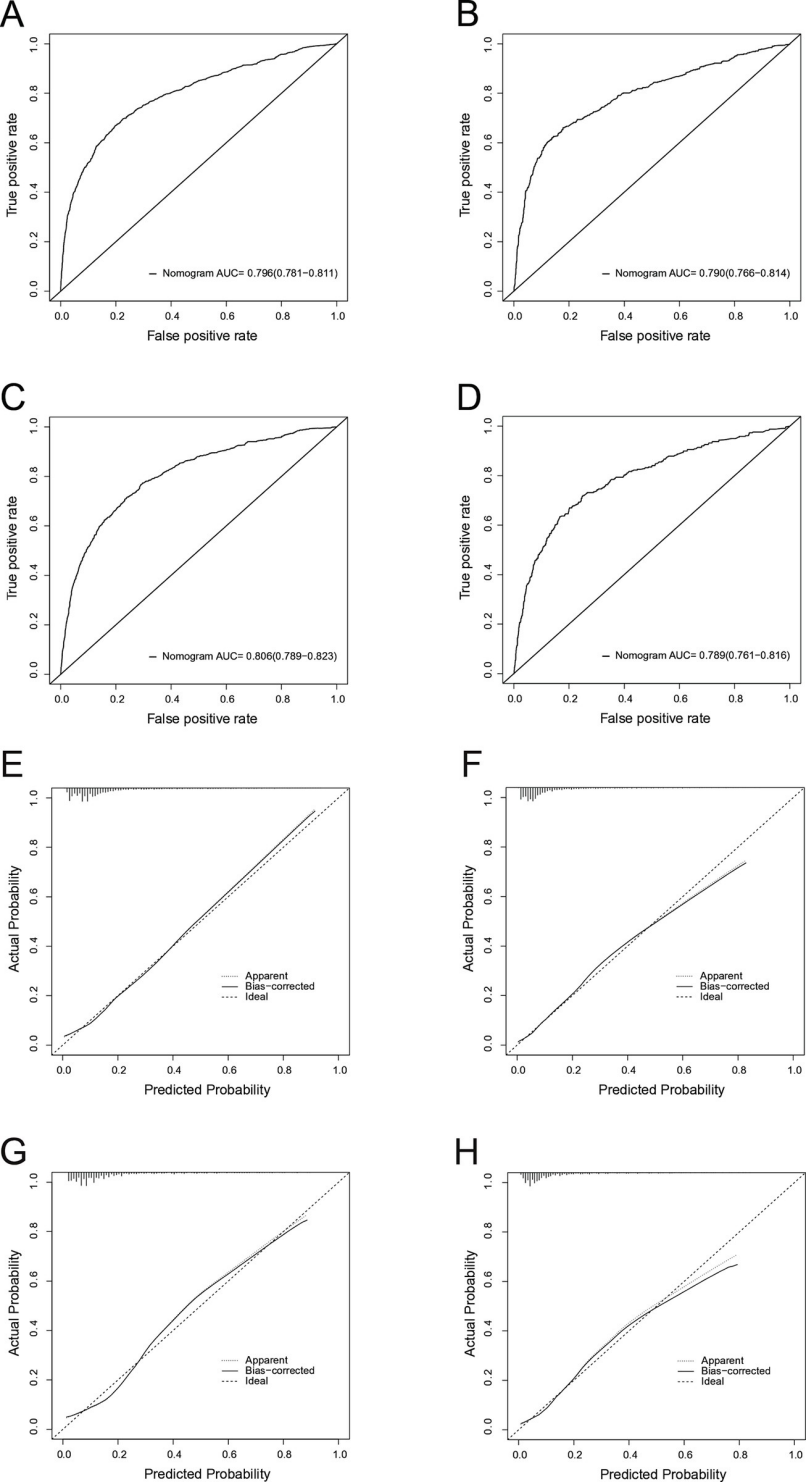
ROC curves for discrimination of the nomograms in predicting all-cause and cancer-specific early death in the training cohort (A, B) and the validation cohort (C, D). Calibration curves for assessing the calibration of the nomogram in predicting all-cause and cancer-specific early death in the training cohort (E, F) and the validation cohort (G, H).

The nomogram plots in the training cohort demonstrated good predictive performance for all-cause and cancer-specific early mortality risk, with AUC values of 0.796 and 0.790, respectively (Fig 3A and 3B). Similarly, in the validation cohort, the AUCs of the nomogram plots for all-cause and cancer-specific early mortality prediction were 0.806 and 0.789, respectively (Fig 3C and 3D). The nomograms exhibited reliable predictability for early mortality events in both nomograms. The calibration curves in Fig 3E–3H further displayed a high degree of agreement between the predicted and actual probabilities of early mortality in the training and validation cohorts, serving as an evaluation of the model’s calibration.

### Clinical applicability

**Fig 4:**
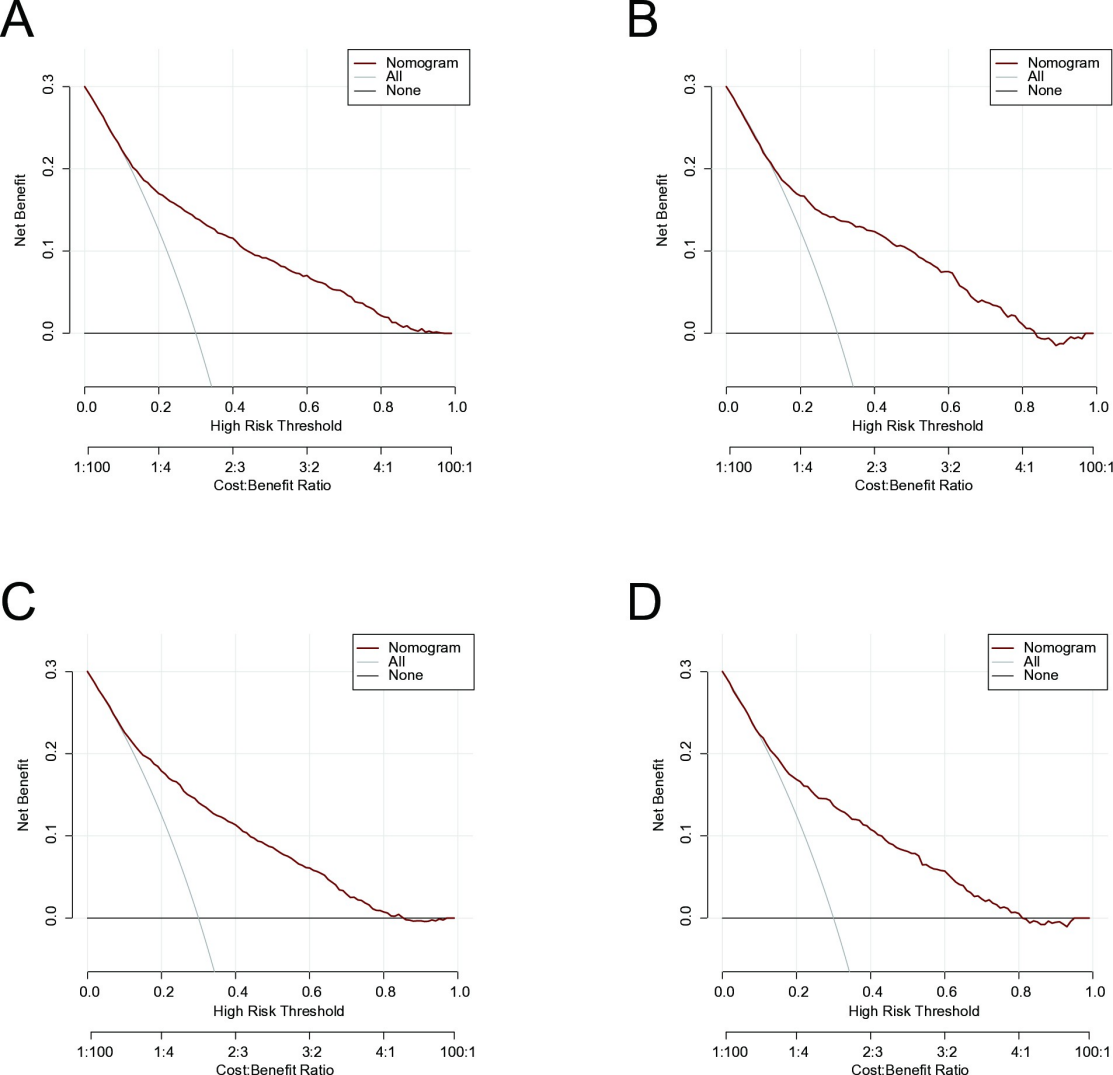
DCAs for the nomograms in predicting all-cause and cancer-specific early death in the training cohort (A, B) and the validation cohort (C, D).

Methods analyzing the cumulative clinical benefit of the prediction model were used to evaluate the nomograms’ clinical applicability. The most favorable threshold probability for predicting early mortality in the training cohort was 0.1–0.8, suggesting that nomograms could help clinicians accurately assess early mortality in elderly patients with LSCC (Fig 4A and 4B).

**Fig 5:**
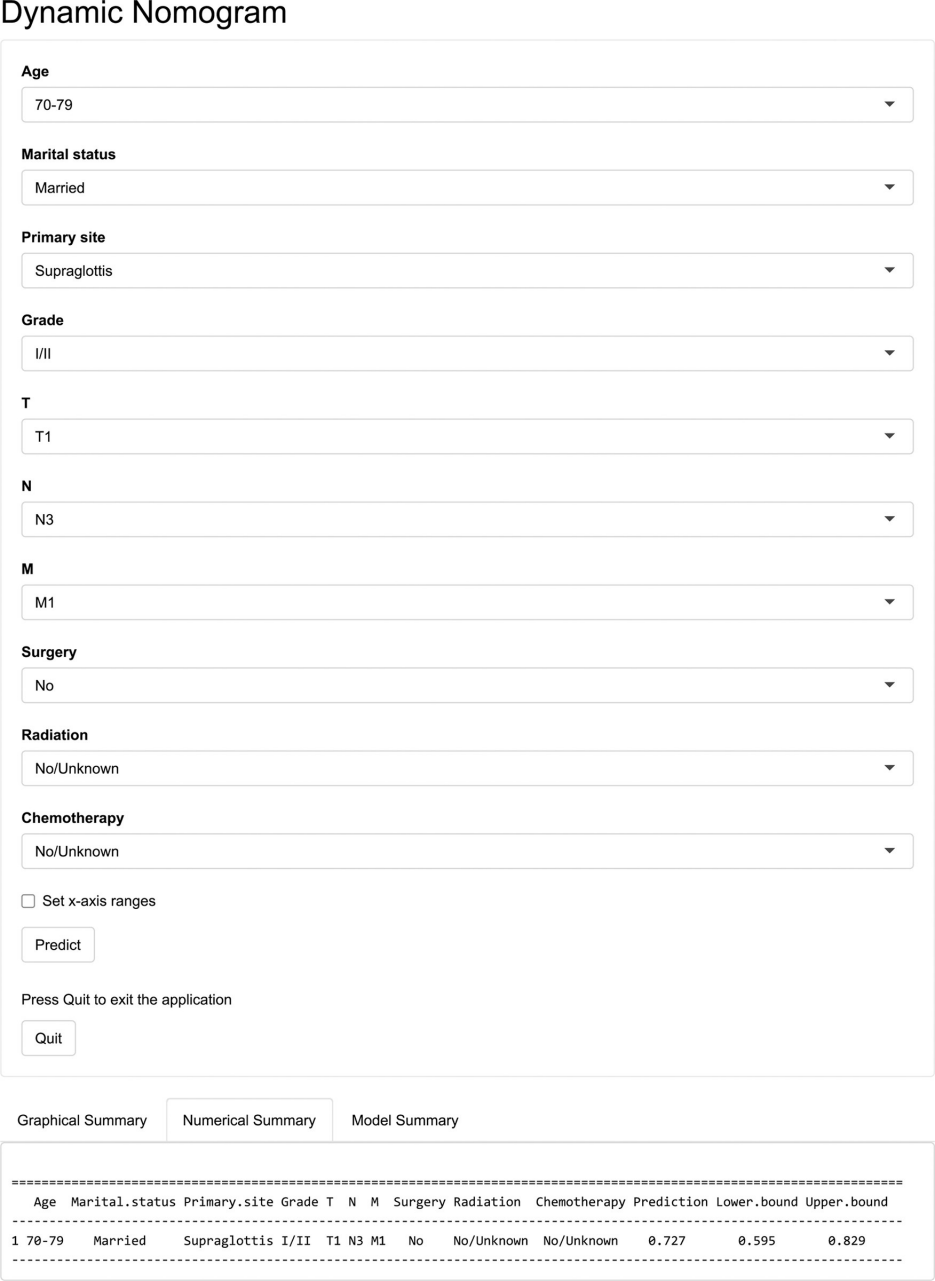
Web-based probability calculator for predicting all-cause early death in elderly LSCC patients.

Two dynamic web-based probability calculators were developed that estimated all-cause early death (https://lqwshiny.shinyapps.io/DynNomapp-OS/) and cancer-specific early death (https://lqwshiny.shinyapps.io/DynNomapp-CSS/) (Fig 5).

## Discussion

The incidence and mortality of LSCC increase with age, peaking at 60 years of age, followed by a plateau [6]. As the population ages, the number of elderly patients with laryngeal cancer is expected to increase. Elderly LSCC patients with compromised health are more likely to experience early mortality compared with younger patients. Similar findings have been reported in other studies investigating various cancer types, where advanced age has been identified as a significant predictor of early death [16, 17]. T The larynx has a distinct anatomical location and plays a crucial role in essential functions such as breathing and eating. Elderly patients with laryngeal cancer face an increased risk of mortality owing to rapid local cancer progression. This could help explain why the 5-year survival rate for laryngeal cancer has been declining, particularly among the elderly and those with advanced stages of the disease, over the past 20 years [6, 7]. Thus, a detailed investigation of the factors associated with early mortality in elderly patients with LSCC is of clinical significance. Herein, the prognostic factors associated with early mortality in elderly patients with LSCC were identified, providing valuable insights for identifying high-risk elderly patients who may require intensified treatment strategies to improve survival outcomes. This is the first study to construct a nomogram to predict early mortality in elderly patients with LSCC.

In terms of demographics, older age (age ≥70 years) and unmarried status were independent risk factors for early mortality in older individuals with LSCC, which is consistent with the previous findings [18–20]. Among all-cause early deaths within the same age group, the proportion of cancer-specific deaths was 57.1% in patients with LSCC aged 60–70 years compared with 69.2% in patients aged ≥70 years, with a significant difference between the two groups. In the nomogram, age occupied about 35 points out of 100-point scale, which is only higher than marital status and tumour grade, with aged 60–70 years being scored 0 and aged ≥80 scored about 35 points. The risk of early death increases with age, which may be attributed to poor physical condition or nutritional status, making them more susceptible to complications and mortality during treatment [21, 22]. Furthermore, marital status affected the survival of patients with LSCC. Married patients with LSCC demonstrated a significantly better prognosis compared with unmarried patients. This is because married patients are more likely to be diagnosed and treated due to “spousal surveillance” and receive better care [23, 24].

Regarding the oncological features, several risk factors, including overlapping lesions of the larynx, advanced T and N stages, distant metastases, and grades III and IV disease, were associated with a high risk of early mortality in elderly patients with LSCC. In the nomogram, TNM stage is closely related to early death. The higher the stage, the higher the chance of early death. Elderly patients with laryngeal cancer have an increased risk of advanced cancer or distant metastasis due to a long history of alcohol and tobacco exposure or a delayed diagnosis [11]. The prognosis of patients in stage III and IV is usually poor because the increase in tumor burden leads to decreased physical function and increased complications [25–27]. In addition, elderly patients with advanced LSCC are more likely to receive non-standardized treatment, resulting in ineffective control of the cancer and recurrence [28]. Furthermore, the incidence of supraglottic cancer and overlapping lesions in the larynx is significantly higher in elderly patients compared with younger patients [29, 30]. In our study, previous cancer histories were considered because of the increasing number of cancer survivors in the past decade [31]. The results revealed that previous cancer history was not a relevant prognostic factor for early death in elderly patients with LSCC. This suggests that previous cancer history might not accelerate death in elderly patients with LSCC, and they should not be discouraged from receiving appropriate treatment based solely on their previous cancer history. However, it is worth noting that some studies have demonstrated that older patients with LSCC could receive more intensive treatment and achieve similar response and survival outcomes compared with younger patients [32–34]. Reizenstein et al. reported that undertreatment, rather than age and complications, is more likely to affect the survival and prognosis of patients [35, 36].

Regarding treatment, our findings revealed that radiotherapy, chemotherapy, and surgical treatment are significantly associated with a favorable prognosis. Among these variables, radiotherapy and surgery emerged as the most significant factors. This might be associated with the specificity of LSCC, wherein LSCC progression affects breathing, feeding structures, and certain vital blood vessels and nerves. Thus, the localized regional progression of LSCC often leads to mortality. Studies have reported that even in stage M1 LSCC, intensifying treatment at the primary site can confer survival benefits to patients [37, 38]. Advances in surgical techniques and intensity-modulated radiotherapy have further improved the safety and efficacy of treatment [39–41].

Nomograms are widely used as prognostic models with high reliability and stability, offering valuable assistance to clinicians in identifying patients requiring immediate care, designing clinical trials, and individualizing treatment modalities [42]. The AUC values of the nomograms in our investigation were approximately 0.8 in the training and validation cohorts, demonstrating satisfactory predictive power. Furthermore, DCA was used to evaluate the efficacy of the prediction model [43], which confirmed its strong predictability and clinical applicability. The inclusion of a nomogram-based network probability calculator further facilitated clinical decision-making. Overall, our results revealed that active treatment, particularly regional treatment of the primary site, could lower early mortality in elderly patients with LSCC. For elderly patients at high risk of early death from LSCC, there is a need for improved treatment strategies and a reduction in the time between diagnosis and treatment initiation to prevent mortality before receiving proper medical intervention [44, 45].

A Despite several studies investigating factors influencing the prognosis of elderly patients with laryngeal cancer, the development of predictive models for survival prediction remains limited [46, 47]. While Pan et al. developed a nomogram to predict lymph node metastasis in elderly patients, they did not analyse survival and prognosis outcomes [48]. Liu et al. analysed patients with supraglottic carcinoma and identified advanced age, male sex, advanced T and N stages, distant metastases, and lack of treatment as factors associated with early death [49]. This is similar to our study, except for the following differences. First, stepwise backward regression logistic analysis was employed to eliminate multicollinearity between variables, which was not addressed in Liu et al’s study. Second, our study focused on elderly patients with LSCC, a condition of high clinical significance due to its high morbidity and mortality. Additionally, previous cancer history was included as a variable in our analysis. Finally, a relevant line diagram and a network-based dynamic probability calculator were constructed to facilitate clinical applicability.

The current study has several limitations. First, the database used in this study lacks information on crucial factors influencing LSCC survival, including smoking, alcohol consumption, and comorbidities. Second, the SEER database lacks precise information on radiotherapy and chemotherapy regimens, cycles, and doses administered to patients. Third, being a retrospective study based on a database, selection bias was inevitable. Finally, our study only performed internal verification, and external verification and larger-scale prospective research are warranted.

## Conclusion

Our findings revealed that overlapping laryngeal lesions, advanced age, unmarried status, advanced T and N stages, distant metastases, and untreated LSCC, contribute to an increased risk of early mortality. Two nomogram plots were constructed based on the multifactorial logistic regression analysis findings to facilitate accurate prediction of all-cause and cancer-specific early mortality within 6 months in elderly patients with LSCC. Implementing precise and safe topical therapy might lower the incidence of early mortality in patients at a higher death risk.

## Supporting information

S1 Table Baseline characteristics of training and validation cohorts. (DOCX)

S2 Table Univariate logistic regression for analyzing the risk factors for early death. (DOCX)
