# Supplementary material for: Nomogram for predicting early death in elderly patients with laryngeal squamous cell carcinoma: A population-based SEER study
Source: PLoS One. 2024 Dec 19;19(12):e0315102. doi: 10.1371/journal.pone.0315102 (PMC11658474; doi:10.1371/journal.pone.0315102)
Supplement: S1 Table — (DOCX) [file pone.0315102.s001.docx]

**Table S1 Baseline characteristics of training and validation cohorts.**

| Characteristic | Number of patients (%) | | P value |
| --- | --- | --- | --- |
|  | Training cohort  N = 7023 | Validation cohort  N = 3008 |  |
| Age |  |  | 0.719 |
| 60-69 | 3040 (43.3) | 1278 (42.5) |  |
| 70-79 | 2544 (36.2) | 1097 (36.5) |  |
| ≥80 | 1439 (20.5) | 633 (21.0) |  |
| Gender |  |  | 0.210 |
| Male | 5650 (80.4) | 2453 (81.5) |  |
| Female | 1373 (19.6) | 555 (18.5) |  |
| Race |  |  | 0.206 |
| White | 5848 (83.3) | 2492 (82.8) |  |
| Black | 955 (13.6) | 401 (13.3) |  |
| Others | 220 (3.1) | 115 (3.8) |  |
| Marital status |  |  | 0.651 |
| Married | 3676 (52.3) | 1590 (52.9) |  |
| Unmarried | 3347 (47.7) | 1418 (47.1) |  |
| Primary site |  |  | 0.555 |
| Glottis | 3452 (49.2) | 1494 (49.7) |  |
| Supraglottis | 2684 (38.2) | 1165 (38.7) |  |
| Subglottis | 142 (2.0) | 64 (2.1) |  |
| Overlapping lesion of larynx | 238 (3.4) | 90 (3.0) |  |
| Larynx, NOS | 507 (7.2) | 195 (6.5) |  |
| Prior cancer history |  |  | 0.58 |
| No | 5150 (73.3) | 2189 (72.8) |  |
| Yes | 1873 (26.7) | 819 (27.2) |  |
| Grade |  |  | 0.631 |
| Ⅰ/II | 5236 (74.6) | 2257 (75.0) |  |
| Ⅲ/V | 1787 (25.4) | 751 (25.0) |  |
| T stage |  |  | 0.521 |
| T1 | 2468 (35.1) | 1088 (36.2) |  |
| T2 | 1807 (25.7) | 788 (26.2) |  |
| T3 | 1560 (22.2) | 633 (21.0) |  |
| T4 | 1188 (16.9) | 499 (16.6) |  |
| N stage |  |  | 0.368 |
| N0 | 5077 (72.3) | 2191 (72.8) |  |
| N1 | 732 (10.4) | 313 (10.4) |  |
| N2 | 1119 (15.9) | 476 (15.8) |  |
| N3 | 95 (1.4) | 28 (0.9) |  |
| M stage |  |  | 0.500 |
| M0 | 6747 (96.1) | 2899 (96.4) |  |
| M1 | 276 (3.9) | 109 (3.6) |  |
| Surgery |  |  | 0.795 |
| No | 4380 (62.4) | 1867 (62.1) |  |
| Yes | 2643 (37.6) | 1141 (37.9) |  |
| Chemotherapy |  |  | 0.704 |
| No/unknown | 4820 (68.6) | 2105 (70.0) |  |
| Yes | 2203 (31.4) | 903 (30.0) |  |
| Radiotherapy |  |  | 0.189 |
| No/Unknown | 1808 (25.7) | 786 (26.1) |  |
| Yes | 5215 (74.3) | 2222 (73.9) |  |
