# Supplementary material for: Nomogram for predicting early death in elderly patients with laryngeal squamous cell carcinoma: A population-based SEER study
Source: PLoS One. 2024 Dec 19;19(12):e0315102. doi: 10.1371/journal.pone.0315102 (PMC11658474; doi:10.1371/journal.pone.0315102)
Supplement: S2 Table — (DOCX) [file pone.0315102.s002.docx]

**Table S2 Univariate logistic regression for analyzing the risk factors for early death.**

| Variable | All-cause early death | | | Cancer-specific early death | | |
| --- | --- | --- | --- | --- | --- | --- |
|  | OR | 95%CI | P value | OR | 95%CI | P value |
| Age |  |  |  |  |  |  |
| 60-69 | reference |  |  | reference |  |  |
| 70-79 | 1.07 | 0.92-1.23 | 0.376 | 1.10 | 0.93-1.31 | 0.266 |
| ≥80 | 1.58 | 1.35-1.85 | **<0.001** | 1.59 | 1.32-1.92 | **<0.001** |
| Gender |  |  |  |  |  |  |
| Male | reference |  |  | reference |  |  |
| Female | 1.15 | 0.98-1.33 | 0.081 | 1.16 | 0.97-1.39 | 0.115 |
| Race |  |  |  |  |  |  |
| White | reference |  |  | reference |  |  |
| Black | 1.21 | 0.98-1.42 | 0.093 | 1.20 | 0.97-1.47 | 0.088 |
| Others | 1.00 | 0.69-1.44 | 0.989 | 0.99 | 0.64-1.53 | 0.968 |
| Marital status |  |  |  |  |  |  |
| Married | reference |  |  | reference |  |  |
| Unmarried | 1.63 | 1.44-1.85 | **<0.001** | 1.63 | 1.4-1.89 | **<0.001** |
| Primary site |  |  |  |  |  |  |
| Glottis | reference |  |  | reference |  |  |
| Supraglottis | 2.04 | 1.77-2.35 | **<0.001** | 2.10 | 1.77-2.48 | **<0.001** |
| Subglottis | 1.58 | 1.01-2.49 | **0.046** | 2.16 | 1.32-3.52 | **0.002** |
| Overlapping lesion of larynx | 2.74 | 2.02-3.73 | **<0.001** | 2.82 | 1.97-4.03 | **<0.001** |
| Larynx, NOS | 3.65 | 2.95-4.53 | **<0.001** | 3.86 | 3.02-4.93 | **<0.001** |
| Prior cancer history |  |  |  |  |  |  |
| Yes | reference |  |  | reference |  |  |
| No | 1.11 | 0.97-1.27 | 0.142 | 0.92 | 0.78-1.10 | 0.361 |
| Grade |  |  |  |  |  |  |
| Ⅰ/II | reference |  |  | reference |  |  |
| Ⅲ/V | 1.57 | 1.37-1.79 | **<0.001** | 1.59 | 1.36-1.87 | **<0.001** |
| T stage |  |  |  |  |  |  |
| T1 | reference |  |  | reference |  |  |
| T2 | 2.00 | 1.65-2.41 | **<0.001** | 2.29 | 1.80-2.90 | **<0.001** |
| T3 | 3.13 | 2.61-3.76 | **<0.001** | 3.64 | 2.89-4.58 | **<0.001** |
| T4 | 4.12 | 3.41-4.97 | **<0.001** | 5.17 | 4.1-6.53 | **<0.001** |
| N stage |  |  |  |  |  |  |
| N0 | reference |  |  | reference |  |  |
| N1 | 2.03 | 1.68-2.44 | **<0.001** | 2.09 | 1.68-2.6 | **<0.001** |
| N2 | 1.95 | 1.67-2.29 | **<0.001** | 2.09 | 1.74-2.52 | **<0.001** |
| N3 | 4.49 | 2.96-6.8 | **<0.001** | 5.26 | 3.4-8.14 | **<0.001** |
| M stage |  |  |  |  |  |  |
| M0 | reference |  |  | reference |  |  |
| M1 | 4.18 | 3.26-5.34 | **<0.001** | 4.99 | 3.85-6.45 | **<0.001** |
| Surgery |  |  |  |  |  |  |
| No | reference |  |  | reference |  |  |
| Yes | 0.43 | 0.37-0.50 | **<0.001** | 0.39 | 0.33-0.47 | **<0.001** |
| Chemotherapy |  |  |  |  |  |  |
| No/unknown | reference |  |  | reference |  |  |
| Yes | 0.19 | 0.17-0.22 | **<0.001** | 0.22 | 0.19-0.25 | **<0.001** |
| Radiotherapy |  |  |  |  |  |  |
| No/Unknown | reference |  |  | reference |  |  |
| Yes | 0.67 | 0.58-0.78 | **<0.001** | 0.75 | 0.64-0.89 | **<0.001** |
